# Analysis of influencing factors and predictive model construction for platelet transfusion efficacy in hematological patients

**DOI:** 10.3389/fmed.2025.1632042

**Published:** 2025-08-07

**Authors:** Yu Zou, Tianhua Jiang, Yue Fan, Simin Liang, Long Lin, Mao Zheng

**Affiliations:** ^1^Department of Blood Transfusion, Deyang People’s Hospital, Deyang, China; ^2^Department of Clinical Laboratory, Deyang People’s Hospital, Deyang, China

**Keywords:** hematologic patients, nomogram, platelet transfusion refractoriness, predictive modeling, risk factors

## Abstract

**Background:**

This study aimed to systematically analyze the independent risk factors for platelet transfusion refractoriness (PTR) in hematological patients, and to develop and validate a nomogram prediction model, thereby providing scientific evidence for personalized platelet transfusion strategies in clinical practice.

**Methods:**

A retrospective cohort study was conducted involving 363 platelet transfusion episodes in hematological patients who received platelet transfusions at Deyang People’s Hospital between January 2023 and August 2023. Comprehensive clinical data and laboratory parameters were collected. Potential PTR-related factors were initially identified through univariate analysis, followed by multivariate logistic regression to determine independent risk factors. Using Rstudio software, a nomogram prediction model was constructed based on the identified factors. The model’s performance was rigorously evaluated through receiver operating characteristic (ROC) curve analysis, calibration curves, and internal validation using bootstrap resampling (1,000 repetitions) to assess discrimination, calibration, and clinical applicability.

**Results:**

This study retrospectively analyzed 363 platelet transfusion episodes involving 131 hematological patients, the incidence of PTR was 30.85% (112/363). Multivariate logistic regression analysis revealed four independent risk factors for PTR: female gender (OR = 1.876, 95% CI: 1.147–3.067), transfusion frequency ≥ 10 times (OR = 2.552, 95% CI: 1.089–5.981), splenomegaly (OR = 3.170, 95% CI: 1.334–7.534), and antibiotic usage (OR = 2.177, 95% CI: 1.078–4.396) (all *p* < 0.05). The predictive model demonstrated an area under the ROC curve of 0.673 (95% CI: 0.611–0.735), with specificity of 78.1%, sensitivity of 55.4%, Youden’s index of 0.335, and an optimal cutoff value of 0.320. Internal validation confirmed good consistency between predicted probabilities and actual observations.

**Conclusion:**

We successfully developed and validated a PTR prediction model incorporating gender, transfusion frequency, splenomegaly, and antibiotic usage as key risk factors. This model exhibits promising clinical utility and can serve as an objective tool for optimizing individualized platelet transfusion protocols in hematological patients.

## 1 Introduction

With the rapid advancement of modern medical technologies, including the widespread application of high-dose chemoradiotherapy in treating malignancies and leukemia, as well as the increasing adoption of hematopoietic stem cell transplantation, clinical demand for platelet products has shown a significant upward trend. Platelet transfusion has become a crucial clinical intervention for preventing and managing hemorrhagic complications caused by thrombocytopenia or platelet dysfunction (1). However, repeated platelet transfusions may induce alloimmunization in patients, potentially leading to platelet transfusion refractoriness (PTR), a clinically significant complication characterized by inadequate post-transfusion platelet count increments (2). PTR represents a complex clinical phenomenon with multifactorial pathogenesis, which can be broadly categorized into immune and non-immune mechanisms (1). Current evidence suggests that non-immune factors account for approximately 80% of PTR cases, primarily including platelet quality defects, hepatosplenomegaly, febrile conditions, severe infections, medication effects, and disseminated intravascular coagulation. These pathological states compromise transfusion efficacy through either structural platelet damage or accelerated platelet consumption (3, 4). Immunological factors account for approximately 20% of cases, primarily attributed to alloimmune responses triggered by repeated blood transfusions, pregnancy, or transplantation. These responses involve complex immunological mechanisms mediated by various antibodies, including human leukocyte antigen (HLA) antibodies, human platelet antigen (HPA) antibodies, CD36 antibodies, ABO blood group antibodies, autoantibodies, drug-dependent antibodies, and immune complexes (5). The reported incidence of PTR exhibits considerable variability across studies, ranging from 10 to 49% (6). This substantial variation likely reflects diagnostic challenges in PTR identification. Among hematological disorder patients, the incidence of PTR ranges from 5 to 34% (7–9). Notably, in hematopoietic stem cell transplantation recipients, more than 50% of cases fail to achieve the anticipated platelet count increment post-transfusion (10). These data underscore the significantly higher PTR prevalence in hematological populations compared to other patient groups. PTR not only imposes substantial economic burdens but also compromises optimal therapeutic outcomes by delaying critical treatments. Consequently, developing effective strategies to mitigate PTR incidence has emerged as a pressing clinical priority. This retrospective cohort study aims to systematically investigate PTR incidence, identify contributing factors, elucidate underlying mechanisms, and ultimately construct a predictive model visualized through a nomogram. Such a tool would facilitate early identification of high-risk patients and inform personalized platelet transfusion strategies in clinical practice.

## 2 Materials and methods

### 2.1 Study design and methods

This retrospective cohort study was conducted at the Department of Hematology, Deyang People’s Hospital, collecting clinical data from hematological patients who received platelet transfusion therapy between January 2023 and August 2023. The study protocol was approved by the Medical Ethics Committee of Deyang People’s Hospital (Ethical Approval No.: 2023-04-079-K01).

#### 2.1.1 Inclusion criteria

Patients were eligible if they met the following criteria: (1) Diagnosed with hematological disorders according to standard diagnostic criteria; (2) Provided written informed consent after being fully informed about the risks associated with repeated platelet transfusions and agreed to undergo related treatments and laboratory tests; (3) Had complete records of at least two platelet transfusion episodes.

#### 2.1.2 Exclusion criteria

Patients were excluded if they: (1) Had incomplete clinical data or transfusion records; (2) Failed to complete key pre- and post-transfusion laboratory evaluations; (3) Presented with severe hepatic or renal dysfunction or other systemic conditions that could potentially affect platelet function. (4) Exclusion of pediatric patients with hematologic disorders.

### 2.2 Research methodology

#### 2.2.1 Platelet preparation and quality control

All apheresis platelet concentrates were uniformly supplied by Deyang Central Blood Station. The collection, preparation, and storage processes strictly adhered to the national standards outlined in the “Quality Requirements for Whole Blood and Blood Components” (GB18469-2012). Each apheresis platelet unit contained one therapeutic dose (10 U) with a volume of approximately 250–300 mL.

#### 2.2.2 Platelet transfusion protocol

Prior to transfusion, patients underwent ABO and Rh blood typing, irregular antibody screening, and cross-matching tests. All blood products were administered following ABO and Rh(D) compatibility guidelines. The transfusion strategy was individualized according to the WS/T623-2018 “Clinical Use of Whole Blood and Blood Components” standard, with platelet transfusion thresholds determined based on patients’ clinical conditions. All platelet products were apheresis-derived, with hematopoietic stem cell transplant recipients receiving γ-irradiated apheresis platelets.

#### 2.2.3 Outcome measures and data collection

Clinical data were extracted from the hospital’s Blood Utilization Management System and Hospital Information System (HIS). The collected parameters included: (1) Demographic characteristics: gender, age, height, and weight; (2) Clinical features: disease type, pregnancy history, and transfusion frequency; (3) Clinical covariates: splenomegaly, history of transfusion reactions, fever (body temperature ≥ 37.3°C on transfusion day), anemia, bleeding, infection (confirmed by laboratory or imaging findings), antibiotic usage, and pre-transfusion prophylactic medication; (4) Laboratory parameters: irregular antibody screening results, platelet storage duration, occurrence of transfusion reactions, and complete blood count parameters before and after transfusion; (5) Imaging findings: splenomegaly confirmed through radiological examination or physical assessment.

#### 2.2.4 Platelet transfusion response evaluation criteria

The efficacy of platelet transfusion was comprehensively assessed using the Corrected Count Increment (CCI) combined with clinical improvement in hemorrhagic symptoms (11). The diagnostic criteria for PTR were defined as follows (12): CCI values < 4.5 × 10^9^/L at 24 h post-transfusion for two consecutive transfusions, or failure to demonstrate significant improvement in clinical bleeding symptoms/hemorrhagic tendency. The CCI was calculated using the following formula: CCI = [(Post-transfusion platelet count–Pre-transfusion platelet count) × Body Surface Area (BSA)]/Total number of transfused platelets × 100%. BSA (m^2^) was calculated using the Dubois formula: BSA = 0.0061 × Height (cm) + 0.0128 × Weight (kg)−0.01529.

Given the practical challenges in routinely obtaining 1-h post-transfusion platelet counts in clinical settings, this study uniformly collected platelet counts within 24 h post-transfusion for efficacy evaluation.

### 2.3 Statistical analysis

Statistical analyses were performed using SPSS 19.0 and GraphPad Prism 9.5. Categorical variables were presented as frequencies (*n*) and percentages (%), with between-group comparisons conducted using χ^2^ tests or Fisher’s exact tests. Normally distributed continuous variables were expressed as mean ± standard deviation (SD) and analyzed using Student’s *t*-tests, while non-normally distributed continuous variables were reported as median[M (P25, P75)] and compared using Mann–Whitney U tests. Univariate and multivariate logistic regression analyses were employed to identify independent risk factors for platelet transfusion refractoriness. Covariates with *p* < 0.05 in univariate analysis were incorporated into the multivariate logistic regression model. Using Rstudio software with the RMS package, a predictive model for platelet transfusion efficacy was developed based on regression coefficients and constant terms. The predictive performance of the model was evaluated through receiver operating characteristic (ROC) curve analysis, with the area under the curve (AUC) calculated to assess discrimination ability. Model sensitivity and specificity were determined, and the optimal cutoff value was identified by maximizing Youden’s index (YI = sensitivity + specificity−1). Calibration curves were generated to evaluate the agreement between predicted probabilities and observed outcomes. All statistical tests were two-tailed, with *p* < 0.05 considered statistically significant.

## 3 Results

### 3.1 Patient characteristics and baseline analysis

This retrospective study analyzed 363 platelet transfusion episodes in 131 patients with hematological disorders, including 67 males (51.15%) and 64 females (48.85%), with a median age of 53 years (interquartile range: 47–60). During the observation period, platelet transfusion refractoriness (PTR) occurred in 112 transfusion episodes, yielding an overall incidence rate of 30.85% (112/363). Comparative analysis between the transfusion-responsive group (*n* = 251) and refractory group (*n* = 112) revealed statistically significant differences (*p* < 0.05) in several baseline characteristics, including gender distribution, transfusion frequency, pregnancy history, splenomegaly status, antibiotic usage, and pre-transfusion platelet count (PLT). Detailed demographic and clinical characteristics are presented in Table 1. Notably, the refractory group demonstrated significantly higher transfusion frequency, greater prevalence of splenomegaly, and more frequent antibiotic administration compared to the responsive group. These findings suggest that these clinical parameters may serve as potential predictors for platelet transfusion outcomes.

**TABLE 1:** Comparative analysis of baseline characteristics between the two patient groups.

Clinical characteristics	Transfusion frequency (*n* = 363)	Effective (*n* = 251)	Ineffective (*n* = 112)	χ^2^/Z value	*P*-value
**Gender [*n* (%)]**					
Male	180 (49.6)	140 (55.8)	40 (35.7)	12.470	0.001
Female	183 (50.4)	111 (44.2)	72 (64.3)		
**Age [M (P_25_, P_75_)]**					
53 (47, 60)	53 (46, 59)	54 (49, 61)	−1.083	0.279
**Transfusion frequency [*n* (%)]**					
< 10 times	188 (51.8)	147 (58.6)	41 (36.6)	14.956	0.001
≥ 10 times	175 (48.2)	104 (41.4)	71 (63.4)		
**Obstetric history [*n* (%)]**					
Positive	143 (39.4)	83 (33.1)	60 (53.6)	13.637	0.001
Negative	220 (60.6)	168 (66.9)	52 (46.4)		
**Splenomegaly [*n* (%)]**					
Present	27 (7.4)	12 (4.8)	15 (13.4)	8.343	0.005
Absent	336 (92.6)	239 (95.2)	97 (86.6)		
**History of transfusion reactions [*n* (%)]**					
Positive	56 (15.4)	45 (17.9)	11 (9.8)	3.901	0.059
Negative	307 (84.6)	206 (82.1)	101 (90.2)		
**Pre-transfusion prophylaxis [*n* (%)]^#^**					
Administered	9 (2.5)	6 (2.4)	3 (2.6)	–	1.000
Not administered	354 (97.5)	245 (97.6)	109 (97.4)		
**Febrile status [*n* (%)]**					
Febrile	85 (23.4)	58 (23.1)	27 (24.1)	0.043	0.893
Afebrile	278 (76.6)	193 (76.9)	85 (75.9)		
**Hemorrhagic status [*n* (%)]**					
Present	182 (50.1)	122 (48.6)	60 (53.6)	0.764	0.427
Absent	181 (49.9)	129 (51.4)	52 (46.4)		
**Infection status [*n* (%)]**					
Present	217 (59.8)	142 (56.6)	75 (67)	3.477	0.065
Absent	146 (40.2)	109 (43.4)			37 (33)
**Antibiotic usage [*n* (%)]**					
Administered	292 (80.4)	193 (76.9)	99 (88.4)	6.510	0.014
Not administered	71 (19.6)	58 (23.1)	13 (11.6)		
**Irregular antibody screening [*n* (%)]^#^**					
Positive	16 (4.4)	9 (3.6)	7 (6.3)	–	0.274
Negative	347 (95.6)	242 (96.4)	105 (93.7)		
**Transfusion reactions [*n* (%)]**					
Occurred	29 (7.99)	21 (8.37)	8 (7.14)	0.158	0.835
Not occurred	334 (92.01)	230 (91.63)	104 (92.86)		
**Blood group [*n* (%)]**					
A	126 (34.72)	88 (35.06)	38 (33.93)	1.190	0.763
B	66 (18.18)	46 (18.33)	20 (17.86)		
O	152 (41.87)	102 (40.64)	50 (44.64)		
AB	19 (5.23)	15 (5.97)	4 (3.57)		
**Platelet storage duration [*n* (%)] (days)**					
< 1	9 (2.48)	4 (1.59)	5 (4.46)	5.601	0.232
1 ∼ < 2	44 (12.12)	27 (10.76)	17 (15.18)		
2 ∼ < 3	124 (34.16)	92 (36.65)	32 (28.57)		
3 ∼ 4	153 (42.15)	104 (41.43)	49 (43.75)		
> 4	33 (9.09)	24 (9.56)	9 (8.04)		
**Pre-transfusion hematological parameters [M (P_25_, P_75_)]**					
WBC (/ × 10^9^/L)	0.91 (0.43, 3.29)	0.91 (0.40, 3.80)	0.89 (0.47, 2.19)	0.624	0.533
LYM (/ × 10^9^/L)	0.44 (0.23, 0.76)	0.40 (0.20, 0.70)	0.50 (0.30, 0.87)	1.950	0.163
MONO (/ × 10^9^/L)	0.04 (0.01, 0.17)	0.05 (0.01, 0.19)	0.03 (0.01, 0.13)	1.931	0.165
NEUT (/ × 10^9^/L)	0.19 (0.03, 1.17)	0.24 (0.03, 1.33)	0.1 (0.03, 0.85)	1.219	0.223
RBC (/ × 10^12^/L)	2.33 (2.04, 2.70)	2.35 (2.05, 2.74)	2.22 (2.01, 2.62)	1.480	0.139
HB (g/L)	71 (63, 84)	72 (65, 85)	68 (61, 79)	1.881	0.060
PLT (× 10^9^/L)	7 (3, 14)	8 (4, 15)	5 (2, 13)	2.429	0.017

^#^Statistical analysis was performed using Fisher’s exact test (applied when more than 20% of cells had expected frequencies less than 5). “Infection status” indicates that the body has been infected by pathogens and is undergoing antibiotic treatment. “antibiotic usage”–refers to the use of antibiotics before blood transfusion. HB, hemoglobin concentration; LYM, lymphocyte count; MONO, monocyte count; NEUT, neutrophil count; PLT, platelet count; RBC, red blood cell count; WBC, white blood cell count.

### 3.2 Heterogeneity analysis of platelet transfusion efficacy among patients with different hematological disorders

This study encompassed a comprehensive cohort of patients diagnosed with various hematological disorders, including myeloid leukemia, lymphocytic leukemia, lymphoma, multiple myeloma, aplastic anemia, myelodysplastic syndrome, maintenance chemotherapy for malignant tumors, primary thrombocytopenia, and immune thrombocytopenia. Comparative analysis revealed statistically significant intergroup differences in platelet transfusion efficacy (*p* < 0.05). Notably, patients with primary thrombocytopenia demonstrated the lowest transfusion efficacy rate (47.06%), whereas those with multiple myeloma and lymphoma exhibited the highest efficacy rates of 100.00 and 92.30%, respectively. The detailed comparative data are presented in Table 2.

**TABLE 2:** Comparative analysis of platelet transfusion efficacy across different hematological disorders (transfusion efficacy determined by CCI).

Diagnosis	Number of cases	Transfusion frequency	Effective transfusions	Efficacy rate (%)	CCI (× 10^9^/L) [M (P_25_, P_75_)]
Myeloid leukemia	52	151	96	63.58	14.12 (9.07, 19.97)
Lymphocytic leukemia	12	37	29	78.38	10.34 (7.02, 14.02)
Lymphoma	7	13	12	92.30	9.25 (8.22, 13.69)
Aplastic anemia	5	12	9	75.00	14.27 (8.34, 22.53)
Myelodysplastic syndrome	6	12	8	66.67	14.3 (10.36, 18.16)
Multiple myeloma	5	12	12	100.00	8.63 (6.61, 10.55)
Maintenance chemotherapy	18	57	36	63.16	13.15 (9.19, 18.22)
Primary thrombocytopenia	7	17	8	47.06	17.7 (11.63, 52.49)
Immune thrombocytopenia	5	11	9	81.82	27.4 (16.61, 44.53)
Other disorders	14	41	33	80.49	12.67 (9.40, 19.66)
Total	131	363	251	69.15	12.77 (8.88, 18.49)

### 3.3 Factors influencing platelet transfusion efficacy

A preliminary univariate logistic regression analysis was conducted to screen potential influencing factors. The results demonstrated statistically significant associations (*p* < 0.05) between platelet transfusion efficacy and five key variables: female gender, cumulative transfusion frequency (≥ 10 times), pregnancy history, splenomegaly, and antibiotic usage. The detailed statistical outcomes are presented in Table 3.

**TABLE 3:** Univariate logistic regression analysis of factors affecting platelet transfusion efficacy.

Variable	β coefficient	Standard error (SE)	Wald χ^2^	*P*-value	OR (95% CI)
Female gender	0.781	0.234	11.172	0.001	2.184 (1.381 ∼ 3.453)
Age	0.070	0.007	0.847	0.357	1.007 (0.992 ∼ 1.022)
Transfusion ≥ 10 times	0.895	0.234	14.597	0.001	2.448 (1.546 ∼ 3.874)
Pregnancy	0.866	0.232	13.895	0.001	2.378 (1.508 ∼ 3.750)
Splenomegaly	1.125	0.406	7.693	0.006	3.080 (1.391 ∼ 6.820)
History of transfusion reactions	−0.696	0.358	3.788	0.052	0.499 (0.247 ∼ 1.005)
Pre-transfusion prophylaxis	0.303	0.739	0.168	0.682	1.354 (0.318 ∼ 5.767)
Fever	0.055	0.267	0.043	0.835	1.057 (0.626 ∼ 1.783)
Hemorrhage	0.199	0.228	0.763	0.382	1.220 (0.781 ∼ 1.906)
Infection	0.442	0.238	3.454	0.063	1.556 (0.976 ∼ 2.480)
antibiotic usage	0.828	0.331	6.263	0.012	2.289 (1.197 ∼ 4.377)
Irregular antibody screening	0.584	0.517	1.273	0.259	1.793 (0.650 ∼ 4.941)
Transfusion reactions	−0.171	0.432	0.157	0.692	0.842 (0.361 ∼ 1.964)
Blood type	−0.482	0.595	0.656	0.418	0.618 (0.192 ∼ 1.983)
Platelet storage duration	0.084	0.125	0.455	0.500	1.088 (0.852 ∼ 1.389)
WBC	0.009	0.009	1.099	0.294	1.009 (0.992 ∼ 1.026)
LYM	−0.205	0.179	1.308	0.253	0.815 (0.573 ∼ 1.158)
MONO	−0.081	0.092	0.765	0.382	0.922 (0.769 ∼ 1.106)
NEUT	0.016	0.029	0.313	0.576	1.017 (0.960 ∼ 1.077)
RBC	0.155	0.159	0.952	0.329	1.167 (0.856 ∼ 1.593)
HB	0.009	0.006	2.303	0.129	1.009 (0.997 ∼ 1.022)
PLT	0.009	0.011	0.726	0.394	1.009 (0.988 ∼ 1.031)

### 3.4 Multivariate regression analysis of platelet transfusion efficacy

Based on the statistically significant variables identified in univariate analysis (*p* < 0.05), we conducted multivariate logistic regression analysis incorporating five potential predictive factors: gender, transfusion frequency, splenomegaly status, antibiotic usage, and pregnancy history. The multivariate analysis revealed that female gender (OR = 1.876, 95% CI: 1.147–3.067), high transfusion frequency (≥ 10 times, OR = 2.552, 95% CI: 1.089–5.981), splenomegaly (OR = 3.170, 95% CI: 1.334–7.534), and antibiotic administration (OR = 2.177, 95% CI: 1.078–4.396) emerged as independent risk factors for platelet transfusion refractoriness (PTR) (*p* < 0.05). Notably, pregnancy history did not demonstrate statistical significance in the multivariate model (*p* = 0.599). The detailed results are presented in Table 4.

**TABLE 4:** Multivariate logistic regression analysis of factors influencing platelet transfusion efficacy.

Variable	β coefficient	Standard error (SE)	Wald χ^2^	*P*-value	OR (95% CI)
Female gender	0.629	0.251	6.281	0.012	1.876 (1.147 ∼ 3.067)
Transfusion ≥ 10	0.937	0.435	4.647	0.031	2.552 (1.089 ∼ 5.981)
Pregnancy	−0.234	0.446	0.277	0.599	0.791 (0.330 ∼ 1.895)
Splenomegaly	1.154	0.442	6.825	0.009	3.170 (1.334 ∼ 7.534)
Antibiotic usage	0.778	0.358	4.712	0.030	2.177 (1.078 ∼ 4.396)

### 3.5 Development and validation of a predictive model for PTR in hematological patients

**FIGURE 1:**
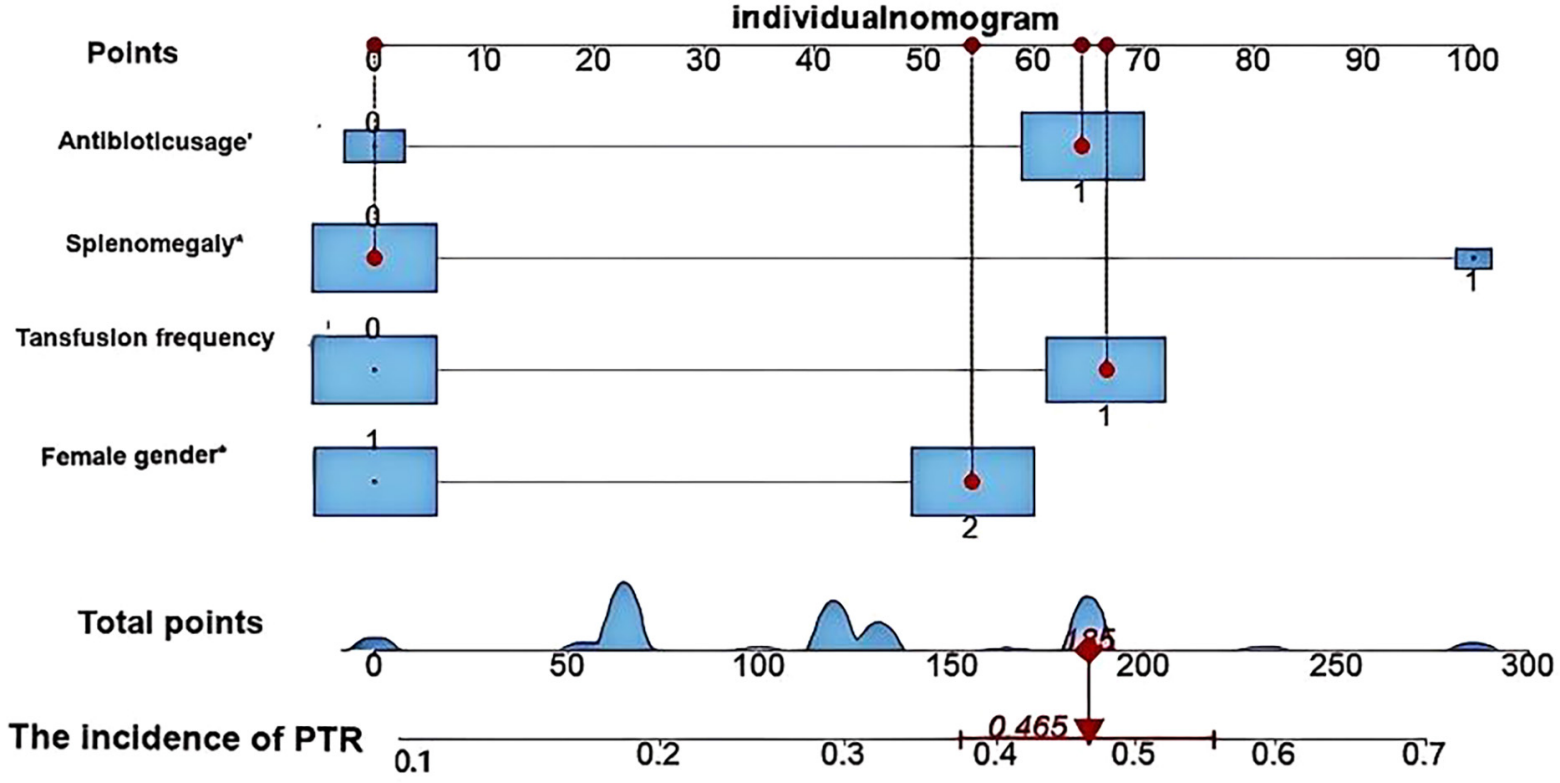
Nomogram model for predicting platelet transfusion efficacy in hematological patients developed using RStudio software. This nomogram illustrates the predictive outcome for a representative case. The patient was female with no history of splenomegaly, had received more than 10 previous platelet transfusions, and was undergoing antibiotic therapy. Based on the model calculation, the patient’s total score was 185 points, corresponding to a 46.5% probability of PTR. These predictive results provide quantitative evidence to support clinical decision-making regarding platelet transfusion.

**FIGURE 2:**
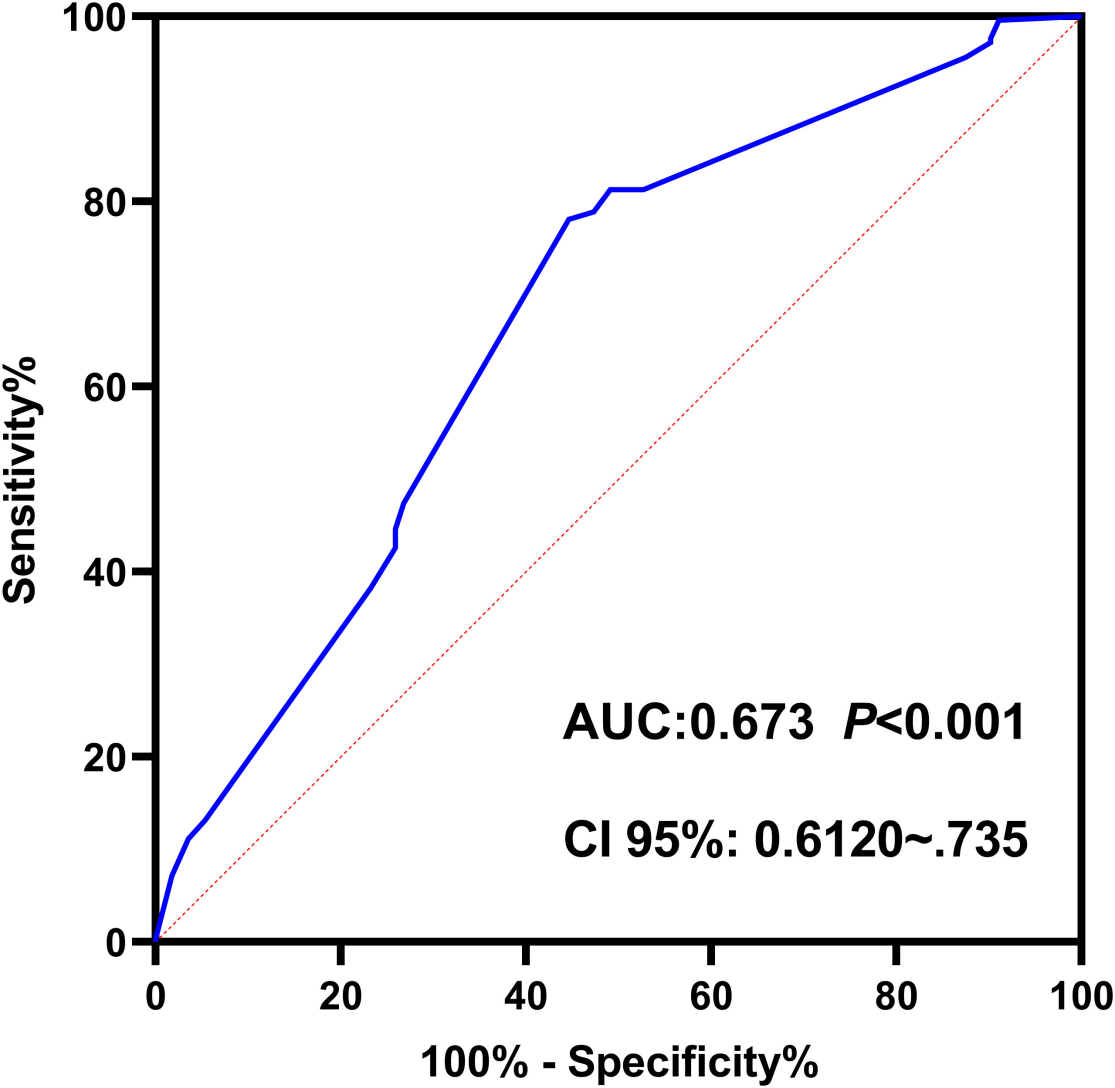
Receiver operating characteristic (ROC) curve of the nomogram-based predictive model for platelet transfusion efficacy in hematological patients. The ROC curve demonstrates the trade-off between sensitivity (*y*-axis) and 1-specificity (*x*-axis). The area under the curve (AUC) of 0.673 indicates moderate discriminative ability of the predictive model.

**FIGURE 3:**
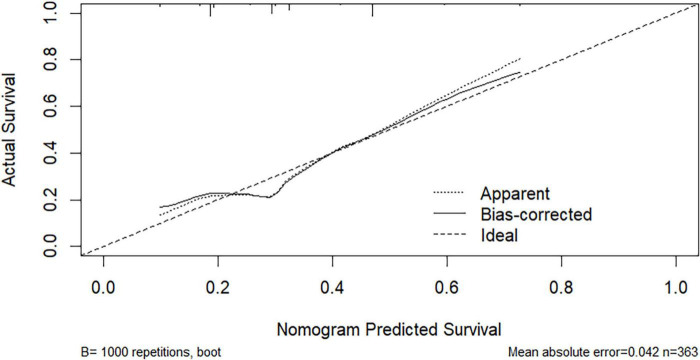
Calibration curve of the fitted nomogram prediction model.

Based on multivariate logistic regression analysis, this study identified four independent risk factors for PTR: gender, transfusion frequency ≥ 10 times, splenomegaly, and antibiotic use. The mathematical expression of the predictive model was formulated as: Logit *P* = 1/{1 + exp[−(−2.277 + 0.629 × gender + 0.937 × transfusion frequency ≥ 10 + 1.154 × splenomegaly + 0.778 × antibiotic use)]}. Utilizing the RStudio software platform, we successfully developed a nomogram prediction model for PTR risk assessment (Figure 1). Model validation demonstrated moderate predictive performance, with an area under the ROC curve (AUC) of 0.673 (95% CI: 0.611–0.735, *p* < 0.05). At the optimal cutoff value of 0.320, the model achieved a specificity of 78.1% and sensitivity of 55.4%, with a Youden’s index of 0.335 (Figure 2). The Hosmer–Lemeshow goodness-of-fit test yielded a statistically significant *P*-value (< 0.05), while the Nagelkerke pseudo R-squared coefficient was computed as 0.128, indicating modest explanatory power. To further validate model reliability, internal validation was performed using the Bootstrap resampling method (1,000 iterations). Calibration curve analysis revealed excellent agreement between predicted probabilities and observed values (Figure 3). The developed nomogram prediction model provides a quantitative tool for clinical assessment of PTR risk in hematological patients, facilitating the formulation of individualized platelet transfusion strategies. This model represents a significant advancement in optimizing transfusion therapy for patients with hematological disorders.

## 4 Discussion

Platelet transfusion serves as a critical supportive therapy for patients with hematological disorders and malignancies undergoing chemotherapy, radiotherapy, or hematopoietic stem cell transplantation, with its clinical efficacy directly impacting treatment outcomes and prognosis. Research indicates that persistent platelet transfusion refractoriness (PTR), if not effectively managed, not only significantly compromises patients’ quality of life but may also pose life-threatening risks. Currently, PTR has emerged as a pressing clinical challenge requiring urgent resolution (13). However, studies investigating specific risk factors for PTR in hematological patients remain limited, and there is a notable lack of reliable predictive models (14, 15). This study employed multivariate regression analysis to identify independent risk factors and subsequently developed a nomogram-based predictive model, thereby providing a theoretical foundation for personalized platelet transfusion strategies.

Our findings revealed a PTR incidence rate of 30.85% among hematological patients, consistent with previous reports (8, 14, 16). This clinical phenomenon not only adversely affects therapeutic efficacy but also presents substantial challenges for optimal healthcare resource allocation. The study identified four independent risk factors significantly associated with PTR: female gender, transfusion frequency ≥ 10 times, splenomegaly, and antibiotic usage. These findings align with multiple published studies (9, 16–19), and offer valuable insights into the pathogenesis of PTR. The underlying mechanisms may involve the following aspects: First, the elevated PTR risk in female patients may be attributed to immunological characteristics. Existing evidence suggests that females exhibit greater susceptibility to immune dysregulation and autoimmune disorders, while pregnancy may induce HLA antibody production–factors that collectively increase the likelihood of platelet alloimmunization (20, 21). This study also identified pregnancy as a potential predictor. While pregnancy showed significance in the univariate model, it did not retain statistical significance in the multivariate model, possibly due to confounding factors such as gender or transfusion frequency. Further investigation is warranted to clarify this relationship. Second, multiple transfusions significantly elevate PTR incidence. Our data demonstrate a dose-dependent relationship between transfusion frequency and PTR risk, with patients receiving ≥ 10 transfusions showing markedly higher PTR rates than those with fewer transfusions. This phenomenon likely results from cumulative exposure to platelet antigens, leading to increased production of HLA class I antibodies and platelet-specific antibodies. These antibodies form immune complexes with transfused platelets, which are subsequently recognized and cleared by splenic macrophages via Fcγ receptor-mediated phagocytosis, ultimately accelerating platelet destruction (2, 16). Third, splenomegaly exerts substantial influence on platelet survival. Multiple studies (9, 22), have documented that splenomegaly impairs post-transfusion platelet increments and shortens transfusion intervals. The enlarged spleen not only sequesters greater quantities of platelets but also enhances the phagocytic activity of the monocyte-macrophage system, collectively reducing circulating platelet counts (18, 16). Finally, antibiotic administration demonstrates significant correlation with PTR occurrence. Platelet-bacterial interactions can promote platelet activation and aggregation through multiple mechanisms (24), including indirect binding via plasma proteins or direct bacterial protein-platelet receptor interactions. Antibiotics may affect platelet transfusion efficacy through two potential pathways: by modulating the host’s immune response status, and by potentially inducing serum antibodies that increase platelet destruction risk (23).

Methodologically, this study innovatively employs a nomogram model to visually present the results of logistic regression analysis. As an intuitive risk prediction tool, nomograms have been widely adopted in clinical research (24–28). Utilizing the RStudio software platform, we integrated four independent risk factors for platelet transfusion refractoriness (PTR) identified through multivariate logistic regression analysis to construct a nomogram-based predictive model. This model demonstrates several notable advantages: (1) It enables intuitive and precise calculation of PTR probability based on individual patient characteristics and risk factor levels; (2) Model validation revealed an area under the ROC curve (AUC) of 0.673 and a Youden index of 0.335, indicating satisfactory discriminative ability. While the model demonstrated statistically significant predictive performance (AUC = 0.673), this represents only moderate predictive capability. The model’s discriminative power has notable limitations, though its performance could potentially be enhanced through external validation; (3) Internal validation through 1000 bootstrap resampling iterations demonstrated excellent agreement between predicted probabilities and observed values in calibration curves, further confirming the model’s predictive efficacy. These features establish the model as an objective, quantitative risk assessment tool for clinicians, facilitating early identification of high-risk PTR patients and implementation of targeted interventions. The model’s reliance on routine clinical parameters enhances its applicability in primary healthcare settings. However, the relatively low sensitivity may impact early detection of high-risk cases, suggesting potential for improvement through incorporation of immunological markers (e.g., HLA antibody screening) in future iterations.

The study’s innovative contributions are threefold: (1) Development of a predictive model based on routinely available clinical parameters, ensuring practical clinical utility; (2) Provision of quantitative evidence to support personalized platelet transfusion strategies; (3) Formulation of differentiated intervention approaches for patients with distinct risk profiles: matched platelet transfusions are recommended for female patients and those requiring long-term multiple transfusions (29–33); splenectomy or primary disease treatment may benefit patients with splenomegaly to address hypersplenism; and antibiotic-treated patients should receive platelet transfusions only after infection control. These personalized interventions are expected to significantly enhance transfusion efficacy and optimize blood resource utilization. Nevertheless, several limitations warrant consideration: (1) The single-center retrospective design may introduce selection bias, while the limited sample size, particularly in subgroup analyses, may compromise statistical power; (2) Exclusion of immunological parameters such as HLA/HPA antibodies may have omitted significant predictive factors; (3) The model’s generalizability may be constrained by its development using data from a single institution and retrospective study design, potentially affecting predictive accuracy and external validity. Therefore, multicenter external validation studies are imperative to further assess the model’s robustness and clinical applicability.

## 5 Conclusion

This study identifies gender, transfusion frequency (≥ 10 times), splenomegaly, and antibiotic use as key non-immunological factors associated with PTR in hospitalized hematological malignancy patients. The successfully constructed nomogram prediction model demonstrates satisfactory discrimination and calibration, providing clinicians with a practical tool for identifying high-risk PTR populations. Implementation of this model will facilitate precision management of platelet transfusions, representing significant clinical value and scientific importance for advancing personalized therapeutic practices.

## Data Availability

The data analyzed in this study is subject to the following licenses/restrictions; the raw data supporting the conclusions of this article will be made available by the authors, without undue reservation. Requests to access these datasets should be directed to 676091264@qq.com.
